# Interference with xenobiotic metabolic activity by the commonly used vehicle solvents dimethylsulfoxide and methanol in zebrafish (*Danio rerio*) larvae but not *Daphnia magna*

**DOI:** 10.1016/j.chemosphere.2012.03.018

**Published:** 2012-08

**Authors:** Rhiannon M. David, Huw S. Jones, Grace H. Panter, Matthew J. Winter, Thomas H. Hutchinson, J. Kevin Chipman

**Affiliations:** a School of Biosciences, University of Birmingham, Edgbaston, Birmingham B15 2TT, UK; b AstraZeneca Safety, Health and Environment, Brixham Environmental Laboratory, Freshwater Quarry, Brixham, Devon TQ5 8BA, UK; c Centre for Environment, Fisheries and Aquaculture Sciences, Barrack Road, The Nothe, Weymouth, Dorset DT4 8UB, UK; d Imperial College London, Department of Surgery and Cancer, Sir Alexander Fleming Building, South Kensington Campus, London SW7 2AZ, UK

**Keywords:** Xenobiotic metabolism, Cytochromes P450, *Daphnia magna*, Zebrafish, Solvents

## Abstract

Organic solvents, such as dimethylsulfoxide (DMSO) and methanol are widely used as vehicles to solubilise lipophilic test compounds in toxicity testing. However, the effects of such solvents upon innate detoxification processes in aquatic organisms are poorly understood. This study assessed the effect of solvent exposure upon cytochrome P450 (CYP)-mediated xenobiotic metabolism in *Daphnia magna* and zebrafish larvae (4 d post fertilisation). Adult *D. magna* were demonstrated to have a low, but detectable, metabolism of ethoxyresorufin *in vivo* and this activity was not modulated by pre-exposure to DMSO or methanol (24 h, up to 0.1% and 0.05% v/v, respectively). In contrast, the metabolism of ethoxyresorufin in zebrafish larvae was significantly reduced by both solvents (0.1% and 0.05% v/v, respectively) after 24 h of exposure. In zebrafish, these observed decreases in activity towards ethoxyresorufin were accompanied by decreased expression of a variety of genes coding for drug metabolising enzymes (corresponding to CYP1, CYP2, CYP3 and UDP-glucuronyl transferase [UGT] family enzymes), measured by quantitative PCR. Reduction of gene expression and CYP1 enzyme activities by methanol (0.05% v/v) in zebrafish larvae was partially reversed by co-exposure with Aroclor 1254 (100 μg L^−1^). Overall this study suggests that relatively low concentrations of organic solvents can impact upon the biotransformation of certain xenobiotics in zebrafish larvae, and that this warrants consideration when assessing compounds for metabolism and toxicity in this species.

## Highlights

► *Daphnia magna* have a detectable activity towards ethoxyresorufin. ► This activity is unaffected by 24 h exposure to dimethylsulfoxide or methanol. ► EROD activity in zebrafish is inhibited by 24 h exposure to both DMSO and methanol. ► DMSO and methanol exposures (24 h) decreased expression of CYP and UGT genes in zebrafish larvae. ► A maximum solvent concentration of 0.01% v/v is recommended for use where possible in zebrafish.

## 1 Introduction

Biotransformation is an important consideration for ecotoxicity testing, as it is known to be a key modulator of the toxicity and bioaccumulation of many xenobiotics. It is, therefore, essential that qualitative and quantitative data on biotransformation pathways in aquatic organisms be obtained (Livingstone, 1998). Cytochromes P450 (CYPs) are the most important enzyme groups involved in the catalysis of phase I drug metabolism reactions (Buhler and Wang-Buhler, 1998). CYPs have an important role in a range of biological processes including genotoxicity and carcinogenesis (Stegeman and Livingstone, 1998), thus it is important to understand the existence of CYPs and their functionality in aquatic organisms.

The freshwater crustacean *Daphnia magna* is used extensively in aquatic toxicology, being one of the species frequently used in international test guidelines for chemicals (OECD, 2004). Despite this, there is limited information available regarding the nature and function of CYPs in daphnids. Of the data that have been published, *D. magna* are known to metabolise pyrene (Akkanen and Kukkonen, 2003; Ikenaka et al., 2006), and the insecticide toxaphene (Kashian, 2004), the involvement of CYPs in this process was indicated by decreased metabolism of both compounds following treatment with the CYP inhibitor piperonyl butoxide (PBO) (Akkanen and Kukkonen, 2003; Kashian, 2004). In addition, CYP3A2, the gene that encodes testosterone-6-β-hydroxylase, has been identified in *Daphnia pulex* (FleaBase, 2009) and treatment with PBO significantly inhibited this activity (Baldwin and LeBlanc, 1994). Moreover, members of the CYP4 family have been identified in *D. pulex*, including CYP4C3 and CYP4B1, and variation in expression has been linked with environmental concentrations of xenobiotics, including polyphenols (David et al., 2003).

As with *D. magna*, zebrafish (*Danio rerio*) are a well characterised model species, especially in the fields of molecular genetics and developmental biology (Kimmel et al., 1995; Langheinrich, 2003). There is also increasing interest in the use of zebrafish embryos and larvae as a model organism for toxicity testing, ecotoxicology screening, and disease modelling, with a particular focus on drug screening assays (e.g. see Berghmans et al., 2008 and Redfern et al., 2008).

Currently 89 CYP genes have been identified in zebrafish (compared to 57 in human, 102 in mouse and 54 in dog), 42 of which are members of the CYP2 family (Goldstone et al., 2009; Nelson, 2009). The metabolism of a variety of different CYP substrates by zebrafish (specifically adult and larval tissue homogenates) such as ethoxyresorufin and pentoxymethylresorufin have been demonstrated (Chung et al., 2004; Nabb et al., 2006; Reschly et al., 2007; Arukwe et al., 2008). Additionally, CYP3A activity, identified by the detection of a 6β-hydroxylation activity towards testosterone, has also been reported using primary hepatocytes from zebrafish (Reschly et al., 2007), and activities towards ethoxyresorufin, ethoxycoumarin and octyloxymethylresorufin (suggesting CYP1, CYP2 and CYP3 activities, respectively) along with phenolic conjugation activity have also been reported in zebrafish larvae as early as 4 d post fertilisation (dpf. Jones et al., 2010).

In studies of xenobiotic metabolism and toxicity, solvents are often required as vehicles to solubilise lipophilic test compounds. This is especially important in the context of aquatic organism testing. Aquatic organisms effectively bathe in the test compound and any solvent used to solubilise this compound and as such are subject to continuous exposure equivalent to the duration of the test protocol. Of the solvents commonly used in aquatic organism testing, dimethylsulfoxide (DMSO) and methanol are two of the most common. Despite the need, there are concerns regarding potential of solvents to affect test organisms, either by their own toxic action, additive, synergistic or antagonistic interactions with the test substance, or via their ability to alter the bioavailability of the test compound (Hutchinson et al., 2006; Haap et al., 2008). In addition, there are growing numbers of reports of the effects of certain solvents (namely DMSO and methanol) on CYP activities in mammalian systems *in vitro* (Chauret et al., 1998; Busby et al., 1999; Easterbrook et al., 2001).

Consequently in the current study, the effect of DMSO and methanol on EROD (ethoxyresorufin-*O*-deethylase) activity in both organisms was investigated at concentrations often employed as vehicles. In addition, an assessment was made of the effects of these solvents on the expression of genes coding for CYP and UDP-Glucuronyl Transferase (UGT) in zebrafish larvae, as representative genes of both oxidative and conjugative metabolism systems.

## 2 Materials and methods

All chemicals were purchased from Sigma–Aldrich (Poole, UK) unless otherwise stated.

### 2.1 Test organism culture and maintenance

#### 2.1.1 *Daphnia magna*

*D. magna*, derived from a culture at AstraZeneca’s Brixham Environmental Laboratory (Brixham, UK), were maintained in OECD-recommended test media, aerated for a minimum of 24 h before use (containing 0.08 M CaCl_2._2H_2_O, 0.02 M MgSO_4_⋅7H_2_O, 0.035 M NaHCO_3_, 0.005 M KCl; (OECD, 2004), modified to contain selenium (0.002 mg L^−1^; Elendt and Bias, 1990). Cultures were held in a temperature-controlled room at 20 ± 1 °C under a 16 h:8 h light:dark photoperiod with a 20 min dawn dusk transition period. Culture medium was renewed twice weekly and daphnids were fed daily with a concentrated suspension of *Chlorella vulgaris*. All cultures were initiated with third or fourth brood neonates <24 h old. These conditions maintained the daphnids in the parthenogenic reproductive cycle.

#### 2.1.2 Zebrafish

Mixed sex adult WIK (Wild-type India Calcutta) strain zebrafish, approximately 9 months of age, were maintained at a density of 20 animals per 8 L tank in the husbandry unit at The University of Birmingham, at 26 ± 1 °C under a 14 h:10 h light:dark photoperiod (290–300 Lux). Fish were held in a Techniplast aquarium in mesh-filtered (biological filtration using Siprorax Grav ring biological filtration and mechanical filtration to 50 μM; Techniplast, Northampton, UK), sterilised (ultra violet – 30 000 μW s^−1^ cm^−2^), dechlorinated tap water, at a conductance of 480–500 μS and pH 7.4. The fish were fed *Artemia nauplii* and zm200 (150–300 μm) −zm400 (500–800 μm) granulated feed (54% protein adult feed; ZMsystems Ltd., Winchester, UK). Following spawning, fertilised eggs were collected and maintained under the same conditions as adult fish, but at 28 ± 1 °C, in accordance with the developmental staging of Kimmel et al., 1995, until of a suitable age for testing. Under these conditions the zebrafish embryos hatched from the chorion between 2 and 3 dpf.

### 2.2 Exposure of *D. magna*

Briefly, groups of 10 adults (7 d old) were collected and transferred to 100 mL fresh media and incubated with the solvents DMSO (0.002%, 0.02% and 0.1% v/v) or methanol (0.001%, 0.01%, 0.05% v/v) for 24 h. In all cases, an untreated control group was prepared and exposure cultures were maintained under the same culture conditions, as described previously, but without feeding. Three replicate experiments, with two replicate beakers per treatment in each experiment, were conducted.

### 2.3 Exposure of zebrafish larvae

Briefly, 30 zebrafish larvae (3 dpf) were collected and transferred to 40 mL dilution water (obtained from the Techniplast aquarium; The University of Birmingham, UK) in 50 mL Falcon tubes and incubated with DMSO (0.1% or 0.01% v/v, analytical grade; Fisher Scientific, UK), methanol (0.05%, 0.01% or 0.001% v/v, HPLC grade; Fisher Scientific, UK) or 100 μg L^−1^ Aroclor 1254 dissolved in methanol with a final exposure concentration of 0.05% v/v for 24 h at 28 ± 1 °C. In all cases, an untreated control group was prepared and three replicate experiments were conducted.

### 2.4 Fluorescence detection of ethoxyresorufin-*O-*deethylase activity

EROD activity was measured in whole adult daphnids (day 7) or whole zebrafish larvae (96 hpf) by directly measuring their ability to convert 7-ethoxyresorufin (7-ER), *in vivo.* The method used to detect the resultant resorufin was a fluorometric stop assay method based on that of Burke and Mayer (1974) as described by David et al. (2009) with the following specific conditions. Groups of 10 daphnids in culture media or groups of 30 zebrafish larvae in dilution water were incubated with 7-ER (8 μM) and dicumarol (10 μM) for 1, 2, 3, 4 or 5 h or 2, 4, 6, 8 or 10 h respectively.

### 2.5 RNA extraction, synthesis of cDNA, Polymerase Chain Reaction (PCR), and Quantitative PCR (qPCR)

Total RNA was extracted from zebrafish using a Qiagen RNeasy kit, as previously described (Jones et al., 2010), and directed in the manufacturer guidelines for tissue extraction (Qiagen, Crawley, UK). Isolated RNA (500 ng) was used as a template for cDNA synthesis. Briefly, a solution of RNA template, random hexamers (1 μl; Biolabs, Hertfordshire, UK) and nuclease-free water was heated to 65 °C for 5 min. To this solution was added 0.1 M DTT (2 μl), 5 × First strand buffer (5 μl) and Superscript™ II reverse transcriptase (0.5 μl) from the Invitrogen Superscript II kit (Invitrogen, Paisley, UK) together with 25 mM dNTP mix (0.4 μl; Bioline, London, UK). The reaction mixture was incubated at 25 °C for 10 min, 42 °C for 90 min and 70 °C for 15 min in a thermocycler, before storage at −20 °C. The cDNA was quantified by spectrophotometry using the NanoDrop ND-1000.

The validated primers (see Table 1) were utilised in quantitative polymerase chain reaction (qPCR) assays using a Sensimix dT SYBR Green kit (Quantace, Finchley, UK). A mastermix of 50 × SYBR green dye, 2 × Sensimix and deionised water was added to 1 μl of each primer (10 mM) and 250 ng μl^−1^ of cDNA template, with a final volume of 25 μl per well, in a 96 well plate format. Products were amplified and detected using a dissociation protocol, with cycling parameters of 95 °C for 30 s (denaturing step) and 60 °C for 30 s (combined annealing and extension), using an ABI Prism 7000 sequence detection system (Applied Biosystems, USA). The melt curves for all samples were analysed and threshold cycle (Ct) values were recorded for each gene in the linear phase of amplification. Analysis of the data followed the ΔΔCt method (Livak and Schmittgen, 2001). The efficiencies of the qPCR reactions were determined using the LinRegPCR software, as described by Ramakers et al. (2003), in order to meet the requirements of the ΔΔCt method of analysis.

### 2.6 Statistical analysis

Data were assessed for normal distribution and homogeneity of variance by Sharipo-Wilks’ test and Levenes’ test respectively, using SPSS version 16 for Windows (SPSS, v.16). Data that were normally distributed and had equal variances were analysed by one-way ANOVA with a Tukey post hoc test, or by independent samples t-test. Data that did not meet the assumptions required for parametric testing were analysed using a Kruskal–Wallis test and Mann–Whitney U test. All graphs display the mean, ± the Standard Error of the Mean (SEM). Values of *p* < 0.05 were deemed to be significant. A *p*-value of <0.05 and <0.01 are indicated by ^*^ and ^**^ respectively.

Linear regression analysis was used to investigate the significance of the linear relationship with time. A one way ANOVA (*p* < 0.05) was used to identify any statistically significant differences between basal EROD activity and that measured following exposure to DMSO and methanol.

## 3 Results

### 3.1 The effect of solvents on EROD activity measured in *D. magna*

**Fig. 1:**
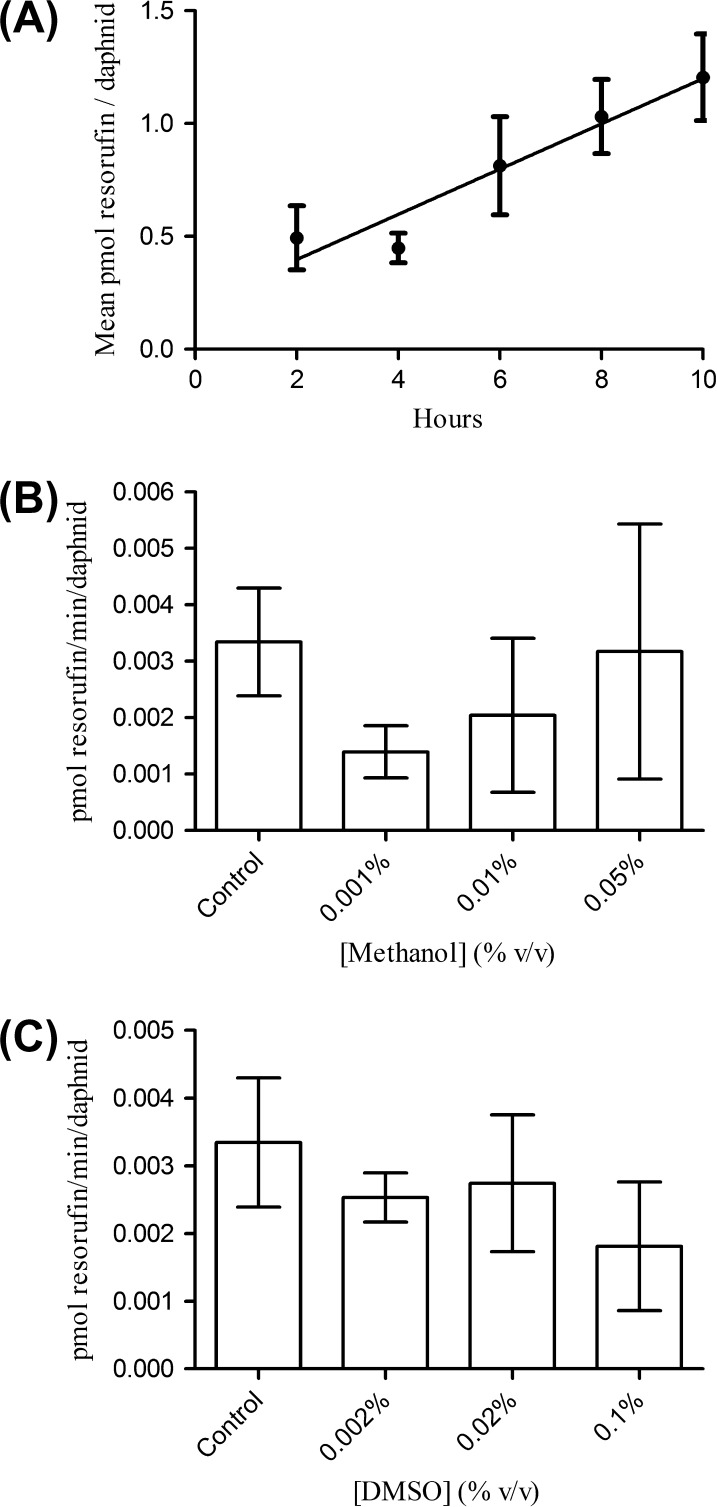
(A) EROD activity measured in 7-d old adult *D. magna*. (B) Effect of methanol pre-exposure (24 h) on EROD activity in *D. magna*. (C) Effect of DMSO pre-exposure (24 h) on EROD activity in adult *D. magna*. All data presented as mean ± SEM for 3 replicate experiments per treatment.

The results suggest that *D. magna* possess measurable CYP1A-like activity. There was a time-dependent increase in the level of resorufin generated, following incubation with 7-ER, and the relationship with time was shown to be close to linearity (*p* > 0.05, *R*^2^ = 0.872; Fig. 1A). No statistically significant concentration-dependent inhibitory effect of DMSO or methanol was detected up to 0.1% and 0.05% respectively (final concentrations 1 mM and 0.5 mM respectively) when administered 24 h prior to measurement of enzyme activity (Fig. 1B and C). It should be noted that enzyme activity was measured in the presence of DMSO as a carrier solvent for the ethoxyresorufin substrate (0.1%, 1 mM).

### 3.2 The effect of solvents on EROD activity and gene expression measured in zebrafish larvae

#### 3.2.1 The effect of methanol on CYP and UGT gene expression

**Fig. 2:**
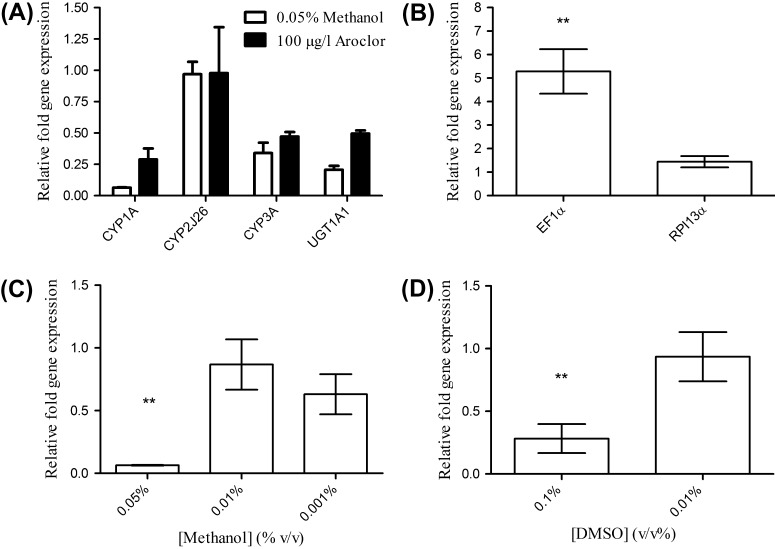
(A) The effect of methanol and Aroclor 1254 pre-exposure (24 h) on CYP and UGT gene expression in zebrafish larvae (4 dpf). (B) Effect of zebrafish larvae (4 dpf) exposed to 0.05% v/v methanol for 24 h prior to determination of EF1α and RPl13α gene expression. (C) Effect on CYP1A expression in zebrafish larvae (4 dpf) exposed to methanol for 24 h. (D) Effect on CYP1A mRNA levels in zebrafish larvae (4 dpf) exposed to DMSO for 24 h. All data presented as mean ± SEM for 3 replicate experiments per treatment. ^*^ and ^**^ represent statistical significance at a level of *p* < 0.05 and *p* < 0.01 respectively.

Pre-exposure with methanol (0.05% v/v, 24 h) significantly reduced the expression of CYP1A to 6% of untreated levels (*p* < 0.05, Mann–Whitney U test). In addition, 0.05% methanol exposure resulted in reduction, although not significant, in the expression of the CYP-gene zgc: 153269 (CYP3A, 34% of untreated levels; *p* < 0.05, Mann–Whitney U test), and UGT1A1 (21% of untreated levels; *p* < 0.05, Mann Whitney U test), but not the expression of CYP2J26, compared to untreated larvae (4 dpf, Fig. 2A). In order to determine if the down-regulation of the assessed genes was due to non-specific repression of gene expression, the effect of methanol pre-treatment (0.05%; 24 h) upon the expression of the validated “reference” genes elongation factor 1α (EF1α) and ribosomal protein l13α (RPl13α) (Tang et al., 2007) was also assessed. Methanol pre-treatment significantly induced EF1α expression (528% increase compared to untreated larvae, *p* < 0.05, Independent samples t-test) but did not alter the expression of RPl13α (Fig. 2B). Taken together, with the lack of modulation of CYP2J26, these results suggest that the reduction in gene expression is a targeted effect of methanol treatment. It was also observed that induction by co-treatment with Aroclor 1254 (100 μg L^−1^) only partially compensated for the loss of expression by methanol of CYP1A (*p* < 0.05, Mann–Whitney U test), and the CYP-gene zgc: 153269 (CYP3A, *p* < 0.05, Mann–Whitney U test) and UGT1A1 (*p* < 0.05, Mann–Whitney U test, Fig. 2A).

#### 3.2.2 Concentration-dependency of the effect of methanol on CYP1A gene expression and EROD activity and compensation by CYP induction

**Fig. 3:**
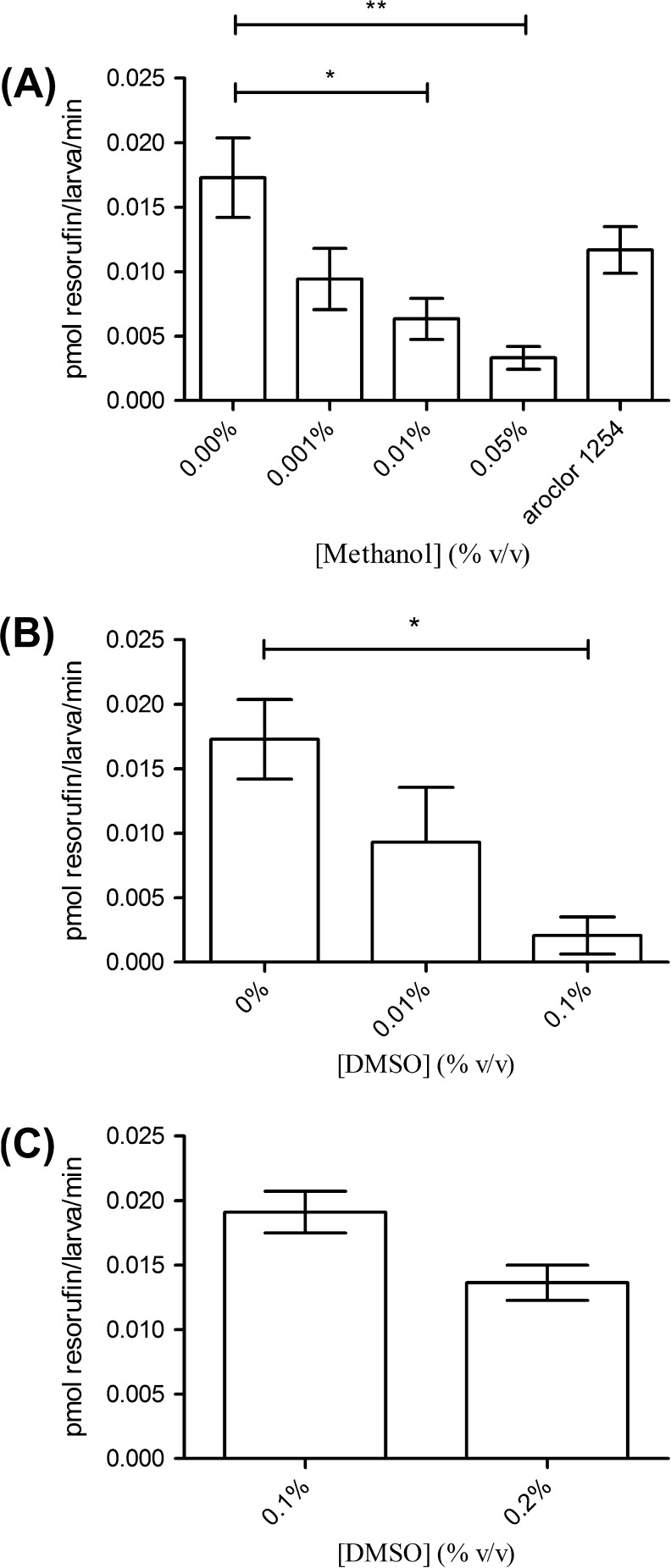
(A) Effect on zebrafish larvae (4 dpf) exposed to methanol, or Aroclor 1254 (100 μg L^−1^) for 24 h before measurement of EROD activity. (B) Effect on zebrafish larvae (4 dpf) exposed to DMSO for 24 h before measurement of EROD activity. (C) EROD activity in zebrafish larvae (4 dpf) exposed to DMSO without a 24 h exposure before assay. As the EROD substrate was dissolved in 0.1% v/v DMSO this was used as the control group. All data is presented as mean ± SEM for 3 replicate experiments per treatment. ^*^ and ^**^ represent statistical significance at a level of *p* < 0.05 and *p* < 0.01 respectively.

The expression of CYP1A was significantly reduced in zebrafish larvae exposed for 24 h to 0.05% v/v methanol, but not at 0.01% before measuring activity (*p* < 0.05, Mann–Whitney U test; Fig. 2C) despite a small but statistically significant inhibition at 0.001%. In contrast, methanol exposure (for 24 h before measuring EROD activity) at 0.01% and 0.05% significantly inhibited EROD activity in the live larval zebrafish assay (Fig. 3A). Co-treatment of zebrafish larvae with Aroclor 1254 was found to recover EROD activity to near-basal levels (Fig. 3A).

#### 3.2.3 The effect of DMSO on CYP1A gene expression and EROD activity

Pre-exposure of zebrafish larvae to 0.1% v/v DMSO (24 h) resulted in a significant reduction in both the expression of CYP1A and EROD activity measured subsequently using 0.1% DMSO as a carrier solvent. These effects were not evident at 0.01% v/v DMSO pre-exposure (Figs. 2D and 3B).

As DMSO had been used regularly to solubilise substrates and compounds such as 7-ER, α-naphthoflavone and SKF-525A at a concentration of 0.1% v/v, the effect of increasing the DMSO vehicle concentration from 0.1% to 0.2% upon EROD activity was assessed. As the ethoxyresorufin substrate is dissolved in DMSO (final concentration 0.1% v/v) this concentration was used as a control in these experiments. The effect of increased solvent concentration without a 24 h incubation before assay was also assessed in these experiments. A slight, but not statistically significant, inhibition of EROD activity was observed after 10 h of elevated DMSO concentration (Fig. 3C).

## 4 Discussion

Despite the widespread use of *D. magna* and zebrafish in ecotoxicity testing and the increased interest in using zebrafish larvae for drug screening, there is relatively limited information regarding the capability of these organisms to metabolise chemicals and on the ability of commonly used vehicle solvents to affect such activities.

In order to assess solvent effects, we firstly determined if CYP activity towards ethoxyresorufin was measureable in *Daphnia*, this itself being of interest in relation to ecotoxicity.

Activity towards ethoxyresorufin was detected in adult *D. magna* (albeit at low levels; peak of 5 × 10^−4^ pmol^−1^ min^−1^ daphnid^−1^) *in vivo* despite a lack of detectable activity reported by Sturm and Hansen (1999) using neonate microsomal preparations. Although Baldwin and Le-Blanc (1994) reported a lack of induction of CYP activity by β-naphthoflavone, a known inducer of CYP1A, another study showed a loss of 20% of administered benzo[a]pyrene following a 24 h incubation (Akkanen and Kukkonen, 2003). Two further studies have shown that *D. magna* are able to metabolise the polycyclic hydrocarbon pyrene (Akkanen and Kukkonen, 2003; Ikenaka et al., 2006). A gene with homology to the novel human CYP2S1, which has been postulated to be important for extrahepatic xenobiotic metabolism (Rylander et al., 2001), has been identified in the *D. pulex* genome, but a search of the database did not reveal any genes with similarity to CYP1A1. In crustaceans it has been shown that substrates of members of the CYP2 and CYP3 families, such as testosterone, are metabolised more rapidly than substrates for CYP1, such as ethoxyresorufin. Moreover, CYP2 and CYP3 family members have been found to be the most abundant CYPs in the hepatopancreas of other crustaceans such as shrimp, lobster, crayfish and crab (James and Boyle, 1998). Although *D. magna* lack a gene homologous to mammalian CYP1A, the presence of other CYP isoforms, combined with the broad-ranging substrate specificities of CYP enzymes and that ethoxyresorufin is metabolised by several CYP isoforms (although predominantly CYP1), probably explains the detected EROD activity. Further investigations into CYP2 and CYP3 isoforms in *D. magna* are also required, as these are the predominant isoforms present in the waterflea and crustaceans.

The effect of the pre-exposure to solvents (up to 0.1% DMSO and 0.05% methanol) on EROD activity was also investigated, but neither solvent affected basal EROD activity in *D. magna*. A concentration of 0.1% DMSO has been reported to inhibit the activity of a number of mammalian recombinant CYPs (Busby et al., 1999), and CYP2E1 in intact human hepatocytes (Easterbrook et al., 2001). Inhibition of CYPs by methanol is less widely reported than for DMSO, and reported inhibition of two CYPs, CYP2E1 and CYP2C9, required higher concentrations (greater than 1%) (Easterbrook et al., 2001) compared to a minimum of 0.2% for inhibition by DMSO (Chauret et al., 1998).

The effect of solvent pre-exposure on EROD activity was also investigated in zebrafish larvae, along with CYP and UGT gene expression (basal EROD activity had previously been detected in zebrafish larvae Jones et al., 2010). Modulation of CYP and UGT gene expression by DMSO (0.1%) and methanol (0.05%) was demonstrated *in vivo*. Interestingly, these concentrations are substantially lower than those often applied to *in vitro* systems, such as cultured hepatocytes or microsomal fractions (⩾0.5% for both solvents). Inhibition of EROD activity by all three concentrations of methanol and 0.1% DMSO was also observed, which agrees with the reported inhibition of CYP1A1 activity in cDNA-expressing B lymphoblastoid cells by these solvents (measuring phenacetin-*O*-deethylase activity). It should be noted that for methanol-exposed larvae, inhibition of EROD activity was observed at 0.01%, a concentration at which there was no significant change in gene expression compared to untreated samples. This suggests that regulation of gene expression is not the only mechanism by which solvent-mediated inhibition of EROD activity occurs. Co-pre-exposure of zebrafish larvae to methanol with the known inducer of CYP gene expression Aroclor 1254 did partially reverse the observed reduction in gene expression and EROD activity. Taken together with the reported inhibition of CYP activities by organic solvents using microsomal fractions (Chauret et al., 1998), the mechanisms of action for this inhibition is unclear.

Thus, despite the fact that zebrafish larvae have been shown to be acutely tolerant to DMSO up to 4% v/v, and that 1% is routinely used in short term drug toxicity assays (e.g. Winter et al., 2008), concentrations of solvents should be minimised to the lowest level possible, especially in longer term tests of, or requiring, CYP-mediated xenobiotic metabolism. In support, OECD guidelines recommend that solvents should be used at a concentration below 0.1 mL L^−1^ (0.01%), which provides a reasonable working concentration for most solvents (OECD, 2000), and minimising concentrations was further supported by Hutchinson et al. (2006) after a review of evidence for the effects of solvents on ecotoxicological test species. In a recent study of DMSO effects on gene expression in zebrafish embryos, altered gene expression in the context of a range of biological processes were also observed at concentrations well below the 1% solvent level often employed in toxicity tests (Turner et al., 2012). This adds further caution to the use of this solvent at such concentrations as a carrier. While the OECD recommended concentration is likely to be suitable for *D. magna,* in view of the apparent inhibitory effects of DMSO and methanol in zebrafish embryo-larvae, where test substance solubility allows, 0.001% v/v is recommended as a maximum concentration in this organism for chronic studies dependent on xenobiotic metabolism. This recommendation is also in accord with a recent recommendation by Chen et al. (2011), that concentrations of ethanol and DMSO as solvent carriers be limited to below 0.01% wherever possible in studies with zebrafish based on effects on behaviour (hyperactivity and altered swimming paths).

## Figures and Tables

**Table 1:** Primers for use for the amplification of gene specific sequences of zebrafish CYP1A, CYP2J26, the CYP-gene zgc: 153269, UGT1A1, AhR1, AhR2, EF1α and RPl13α (validated by sequencing, Jones et al., 2010).

Gene	Left primer	Right primer
CYP1A	TCC TGG AAA TCG GAA ACA AC	CTG AAC GCC AGA CTC TTT CC
CYP2J26	AAG CCC ACA AAA ACC TCC CT	ATA TCA TTG GAT GGG CGG TA
zgc: 153269 (CYP3A)	GGT GGA GGA GAT CGA CAA AA	ACC GTT TTC TTA GCG GAC CT
UGT1A1	CTG CTG GTT GCA TTG AAG AA	CGA TGA CGT CCA GAG AGT GA
RPl13α	TCT GGA GGA CTG TAA GAG GTA TGC	AGA CGC ACA ATC TTG AGA GCA G
EF1α	CTG GAG GCC AGC TCA AAC AT	ATC AAG AAG AGT AGT ACC GCT AGC ATT AC

## References

[b0005] Akkanen J., Kukkonen J.V. (2003). Biotransformation and bioconcentration of pyrene in *Daphnia magna*. Aquatic Toxicology.

[b0010] Arukwe A., Nordtug T., Kortner T.M., Mortensen A.S., Brakstad O.G. (2008). Modulation of steroidogenesis and xenobiotic biotransformation responses in zebrafish (*Danio rerio*) exposed to water-soluble fraction of crude oil. Environmental Research.

[b0015] Baldwin W.S., LeBlanc G.A. (1994). Identification of multiple steroid hydroxylases in *Daphnia magna* and their modulation by xenobiotics. Environmental Toxicology and Chemistry.

[b0020] Berghmans S., Butler P., Goldsmith P., Waldron G., Gardner I., Golder Z., Richards F.M., Kimber G., Roach A., Alderton W., Fleming A. (2008). Zebrafish based assays for the assessment of cardiac, visual and gut function – potential safety screens for early drug discovery. Journal of Pharmacological and Toxicological Methods.

[b0025] Buhler D.R., Wang-Buhler J.-L. (1998). Rainbow trout cytochrome P450s: purification, molecular aspects, metabolic activity, induction and role in environmental monitoring. Comparative Biochemistry and Physiology Part C: Pharmacology, Toxicology and Endocrinology.

[b0030] Burke M.D., Mayer R.T. (1974). Ethoxyresorufin: direct fluorimetric assay of a microsomal o-dealkylation which is preferentially inducible by 3-methylcholanthrene. Drug Metabolism and Disposition.

[b0035] Busby W.J., Ackermann J.M., Crespi C.L. (1999). Effect of methanol, ethanol, dimethyl sulfoxide, and acetonitrile on in vitro activities of cDNA-expressed human cytochromes P-450. Drug Metabolism and Disposition.

[b0040] Chauret N., Gauthier A., Nicoll-Griffith D.A. (1998). Effect of common organic solvents on in vitro cytochrome P450-mediated metabolic activities in human liver microsomes. Drug Metabolism and Disposition.

[b0045] Chen T.-H., Wng Y.-H., Wu Y.-H. (2011). Developmental exposures to ethanol or dimethylsulfoxide at low concentrations alter locomotor activity in larval zebrafish: implications for behavioral toxicity bioassays. Aquatic Toxicology.

[b0050] Chung W.-G., Sen A., Wang-Buhler J.-L., Yang Y.-H., Lopez N., Merrill G.F., Miranda C.L., Hu C.-H., Buhler D.R. (2004). CDNA-directed expression of a functional zebrafish CYP1A in yeast. Aquatic Toxicology.

[b0055] David P., Dauphin-Villemant D., Mesneau A., Meyran C. (2003). Molecular approach to aquatic environmental bioreporting: differential response to environmental inducers of cytochrome P450 monooxygenase genes in the detritivorous subalpine planktonic Crustacea, *Daphnia pulex*. Molecular Ecotoxicology.

[b0060] David R.M., Winter M.J., Chipman J.K. (2009). Induction of DNA strand breaks by genotoxicants in the alga Chlamydomonas reinhardtii. Environmental Toxicology and Chemistry.

[b0065] Easterbrook J., Lu C., Sakai Y., Li A.P. (2001). Effects of organic solvents on the activities of cytochrome P450 Isoforms, UDP-dependent glucuronyl transferase, and phenol sulfotransferase in human hepatocytes. Drug Metabolism and Disposition.

[b0070] Elendt B.-P., Bias W.-R. (1990). Trace nutrient deficiency in *Daphnia magna* cultured in standard medium for toxicity testing. Effects of the optimization of culture conditions on life history parameters of *D. magna*. Water Research.

[b0075] FleaBase, 2009. <http://wfleabase.org/> (accessed May 2009).

[b0080] Goldstone, J., McArthur, A., Zanette, J., Jonsson, M., Parente, T., Stegeman, J.J. 2009. The total cytochrome P450 complement of zebrafish and the expression of CYP genes during development. In: 15th International Symposium on Pollutant Responses in Marine Organisms, Abstract 319.

[b0085] Haap T., Triebskorn R., Köhler H.-R. (2008). Acute effects of diclofenac and DMSO to *Daphnia magna*: Immobilisation and hsp70-induction. Chemosphere.

[b0090] Hutchinson T.H., Shillabeer N., Winter M.J., Pickford D.B. (2006). Acute and chronic effects of carrier solvents in aquatic organisms: a critical review. Aquatic Toxicology.

[b0095] Ikenaka Y., Eun H., Ishizakab M., Miyabara Y. (2006). Metabolism of pyrene by aquatic crustacean, *Daphnia magna*. Aquatic Toxicology.

[b0100] James M.O., Boyle S.M. (1998). Cytochromes P450 in crustacea. Comparative Biochemistry and Physiology Part C: Pharmacology, Toxicology and Endocrinology.

[b0105] Jones H.S., Panter G.H., Hutchinson T.H., Chipman J.K. (2010). Oxidative and conjugative xenobiotic metabolism in zebrafish larvae in vivo. Zebrafish.

[b0110] Kashian D.R. (2004). Toxaphene detoxification and acclimation in *Daphnia magna*: do cytochrome P-450 enzymes play a role?. Comparative Biochemistry and Physiology Part C: Toxicology and Pharmacology.

[b0115] Kimmel C.B., Ballard W.W., Kimmel S.R., Ullmann B., Schilling T.F. (1995). Stages of embryonic development of the zebrafish. Developmental Dynamics.

[b0120] Langheinrich U. (2003). Zebrafish: a new model on the pharmaceutical catwalk. BioEssays.

[b0125] Livak K.J., Schmittgen T.D. (2001). Analysis of relative gene expression data using real-time quantitative PCR and the 2-[Delta][Delta]CT method. Methods.

[b0130] Livingstone D.R. (1998). The fate of organic xenobiotics in aquatic ecosystems: quantitative and qualitative differences in biotransformation by invertebrates and fish. Comparative Biochemistry and Physiology – Part A: Molecular and Integrative Physiology.

[b0135] Nabb D.L., Mingoia R.T., Yang C.-H., Han X. (2006). Comparison of basal level metabolic enzyme activities of freshly isolated hepatocytes from rainbow trout (*Oncorhynchus mykiss*) and rat. Aquatic Toxicology.

[b0140] Nelson D.R. (2009). The cytochrome P450 homepage. Human Genomics.

[b0145] OECD, 2000. OECD Environmental Health and Safety Publications Series on Testing and Assessment No. 23 Guidance Document On Aquatic Toxicity Testing Of Difficult Substances And Mixtures. Paris, France.

[b0150] OECD, 2004. Organization for Economic Cooperation and Development Guidelines for the testing of chemicals number 202: Daphnia sp., Acute Immobilisation Test. Geneva, Switzerland.

[b0155] Ramakers C., Ruijter J.M., Lekanne R.H., Moorman A.F.M. (2003). Assumption-free analysis of quantitative real-time polymerase chain reaction (PCR) data. Neuroscience Letters.

[b0160] Redfern W.S., Waldron G., Winter M.J., Butler P., Holbrook M., Wallis R., Valentin J.-P. (2008). Zebrafish assays as early safety pharmacology screens: paradigm shift or red herring?. Journal of Pharmacological and Toxicological Methods.

[b0165] Reschly E., Bainy A., Mattos J., Hagey L., Bahary N., Mada S., Ou J., Venkataramanan R., Krasowski M. (2007). Functional evolution of the vitamin D and pregnane X receptors. BMC Evolutionary Biology.

[b0170] Rylander T., Neve E.P., Ingelman-Sundberg M., Oscarson M. (2001). Identification and tissue distribution of the novel human cytochrome P450 2S1 (CYP2S1). Biochemical and Biophysical Research Communications.

[b0185] Stegeman J.J., Livingstone D.R. (1998). Forms and functions of cytochrome P450. Comparative Biochemistry and Physiology Part C: Pharmacology, Toxicology and Endocrinology.

[b0190] Sturm A., Hansen P.D. (1999). Altered cholinesterase and monooxygenase levels in *Daphnia magna* and *Chironomus riparius* exposed to environmental pollutants. Ecotoxicology and Environmental Safety.

[b0195] Tang R., Dodd A., Lai D., McNabb W.C., Love D.R. (2007). Validation of zebrafish (Danio rerio) reference genes for quantitative real-time RT-PCR normalization. Acta Biochimica et Biophysica Acta.

[b0200] Turner C., Swale A., Fenske M., Cossins A. (2012). Implications of the solvent vehicles dimethylformamide and dimethylsulfoxide for establishing transcriptomic endpoints in the zebrafish embryo toxicity test. Environmental Toxicology and Chemistry.

[b0205] Winter M.J., Redfern W.S., Hayfield A.J., Owen S.F., Valentin J.-P., Hutchinson T.H. (2008). Validation of a larval zebrafish locomotor assay for assessing the seizure liability of early-stage development drugs. Journal of Pharmacological and Toxicological Methods.

